# Integrated particle image velocimetry and fluid–structure interaction analysis for patient-specific abdominal aortic aneurysm studies

**DOI:** 10.1186/s12938-023-01179-8

**Published:** 2023-12-03

**Authors:** Can Özcan, Özgür Kocatürk, Civan Işlak, Cengizhan Öztürk

**Affiliations:** 1 Institute of Biomedical Engineering, Boğaziçi University, Kandilli Campus, Feza Gürsey Bld., Çengelköy, 34685 Istanbul, Turkey; 2 Department of Radiology, Division of Neuroradiology, Cerrahpaşa Medical Faculty, Istanbul University Cerrahpaşa, Istanbul, Turkey

**Keywords:** Particle image velocimetry, Fluid–structure interaction, 3D printing, AAA

## Abstract

**Background:**

Understanding the hemodynamics of an abdominal aortic aneurysm (AAA) is crucial for risk assessment and treatment planning. This study introduces a low-cost, patient-specific in vitro AAA model to investigate hemodynamics using particle image velocimetry (PIV) and flow-simulating circuit, validated through fluid–structure interaction (FSI) simulations.

**Methods:**

In this study, 3D printing was employed to manufacture a flexible patient-specific AAA phantom using a lost-core casting technique. A pulsatile flow circuit was constructed using off-the-shelf components. A particle image velocimetry (PIV) setup was built using an affordable laser source and global shutter camera, and finally, the flow field inside the AAA was analyzed using open-source software. Fluid–structure interaction (FSI) simulations were performed to enhance our understanding of the flow field, and the results were validated by PIV analysis. Both steady-state and transient flow conditions were investigated.

**Results:**

Our experimental setup replicated physiological conditions, analyzing arterial wall deformations and flow characteristics within the aneurysm. Under constant flow, peak wall deformations and flow velocities showed deviations within − 12% to + 27% and − 7% to + 5%, respectively, compared to FSI simulations. Pulsatile flow conditions further demonstrated a strong correlation (Pearson coefficient 0.85) in flow velocities and vectors throughout the cardiac cycle. Transient phenomena, particularly the formation and progression of vortex structures during systole, were consistently depicted between experimental and numerical models.

**Conclusions:**

By bridging high-fidelity experimental observations with comprehensive computational analyses, this study underscores the potential of integrated methodologies in enhancing our understanding of AAA pathophysiology. The convergence of realistic AAA phantoms, precise PIV measurements at affordable cost point, and validated FSI models heralds a new paradigm in vascular research, with significant implications for personalized medicine and bioengineering innovations.

**Supplementary Information:**

The online version contains supplementary material available at 10.1186/s12938-023-01179-8.

## Background

Abdominal aorta aneurysm (AAA) is a vascular disease that is responsible for 1–2% of elderly man deaths [1–3]. Clinically, it is defined by a localized increase in aortic diameter by more than 50% [1]. There are two alternative treatment options for AAA: endovascular repair and open surgery. Currently, the choice of treatment and rupture risk is primarily assessed by the aneurysm size [4], although more advanced, biomechanics-based prediction systems have been proposed [5].

Endovascular aneurysm repair (EVAR) has been developed since the 1990s. Despite evident benefits (faster recovery and short hospital stay), endovascular solutions have questionable [6] long-term performance (device migration under hypertension [7], endoleak [8], and mechanical failure[9]). Therefore, there is a need to make aneurysm and treatment option studies faster, cheaper, and easier. Democratization of biomedical research instrumentation is a key factor to support numerical analysis and experimental studies based on in vitro phantoms.

In vitro velocity measurements of AAA have been performed by many researchers using particle image velocimetry (PIV) [10–12], 4D magnetic resonance imaging (MRI) [13], and laser Doppler velocimetry [14]; the formation of a vortex ring in the aneurysm sac during the end of systole and its effects on wall shear stresses have been successfully quantified. Boersen et al. [15] studied flow dynamics in EVAR of AAA and presented flow patterns for different endograft designs. Others [16–21] concentrated on developing patient-specific phantoms for optical flow measurements. Geoghegan et al. [22] described the use of the investment casting method for producing MRI and computed tomography (CT)-based phantoms for flow visualization. Yazdi et al. [23] also studied phantom fabrication techniques specific to PIV measurements. They underlined the limitations of the methods for high accuracy measurements as well as the capability of the current state modeling techniques for cardiovascular disease treatment and vascular implant design. Their follow-up study [21] showed a method to produce a thin-walled compliant phantom for PIV measurements. However, their application lacked a trial on a patient-specific geometry.

Many studies [7–9, 24–27] have studied AAA flow dynamics using fluid–structure interactions (FSI). Most recently, Qioa et al. employed FSI method in idealized aorta with aneurysm [28] and in patient-specific healthy aorta [29].

Others [30–34] concentrated on flow-only studies via computational fluid dynamics (CFD), with rigid wall assumption with justification of wall stiffness being high with disease progression [35]. Meyer et al. studied only the structural deformations using image-based measurement techniques using compliant phantoms [36]. More studies that study hemodynamics numerically [7, 8, 24, 25, 27, 37–39] and experimentally [10–12, 14, 15, 21, 39] have opted for the flexible arterial wall assumption. AAA pathology and treatment involve flexible tissue interacting with stent-graft systems and blood. For cases where both fluid and structural parts interact and affect each other, it is crucial to study the AAA two-way coupled FSI [10, 40].

A few numerical [15, 27, 41] and experimental [10, 11, 14] studies on AAA hemodynamics have used idealized geometries, and many [26, 30, 42–45] have decided to analyze patient-specific geometries. The first anatomically accurate vascular models were made using the method of corrosion casting based on human samples [46]. With the advancement of 3D printing and medical image segmentation, medical image-based phantoms have replaced the use of vascular specimens and allowed patient-specific phantom manufacturing [16–18, 22, 43, 47–49]. A substantial number of researchers have used PIV [19, 30, 44] and FSI [24–26, 38] methods to study flow dynamics on patient-specific vascular geometries. There have been individual cost reduction efforts for phantom manufacturing [16, 50], computational frameworks [51], and PIV [52, 53].

In this paper, we present a comprehensive, patient-specific workflow for the experimental and numerical study of AAAs, a methodology not previously reported in the literature. To our knowledge, no validated patient-specific FSI models of AAA exist to date [12]. The most relevant study to our approach involves a thoracic aortic aneurysm (TAA) [39], employing a method that necessitates costly phantom manufacturing and PIV flow measurement systems. Ong et al. studied thoracic aortic aneurysms under a pulsatile flow regime using the FSI analysis with PIV [39]. They were able to capture the formation of a vortex, which may explain aneurysm formation. Our innovative workflow encompasses image-based phantom fabrication, simulation of pulsatile flow circuits, flow analysis utilizing PIV, and detailed numerical analysis through the application of the FSI method. This integrated approach not only advances the study of the AAAs but also proposes a cost-effective and replicable model for further research in this critical area.

## Results

This paper aims to prove that a patient-specific in vitro model of an AAA can be manufactured, and hemodynamics can be investigated using a flow-simulating circuit and an in-house developed PIV system. To determine whether the analysis results obtained from such a workflow are accurate and useful, we compared the arterial wall deformations, flow velocity, flow vectors, and transient vortex formation behavior obtained from PIV of the AAA phantom against a detailed set of FSI simulations. Our results show that the peak wall deformations and flow velocities are within − 12% to + 27% and − 7% to + 5% under constant flow conditions, respectively. Comparing the transient results, we were able to observe good correlation of flow velocities and vectors throughout the cardiac cycle and capture vortex generation within the aneurysm sac.

### Constant flow rate conditions: wall deformations

Blood flow through the aorta is pulsatile and transient in nature; nevertheless, a constant flow rate and steady-state flow analysis allow the comparison of basic quantities between experimental and numerical results. Four different flow rate levels were set and analyzed. The flow rates were chosen to capture expected peak velocity values in transient. The stiffness of the PDMS was calibrated using the wall deformations obtained in this step.

The wall deformations were extracted using the raw PIV image data set (Fig. 8a). Raw PIV images were first processed to extract the outline of the aneurysm wall (Fig. 8b). The average wall displacement amount is then compared against values obtained from two-way steady-state FSI calculations. The peak wall displacements between the experimental and numerical studies match (Table 1) within − 12% and 0% for the flow rate range (0 L/min to 7.2 L/min) of the pulsatile flow. The PDMS material calibration was performed to ensure a good match at the peak flow rate value.

**Table 1:** Comparison of the mean wall displacements calculated by the FSI simulations and measured under constant flow rate conditions

Flow rate (L/min)	Mean δ_experimental_ (mm)	Mean δ_FSI_ (mm)	Relative difference with respect to δ _experimental_ (%)
4.9	0.34	0.30	− 12.29
6.7	0.51	0.51	− 0.39
9.1	0.72	0.89	22.37
11.6	1.11	1.41	27.27

### Constant flow rate: flow velocities

**Fig. 1:**
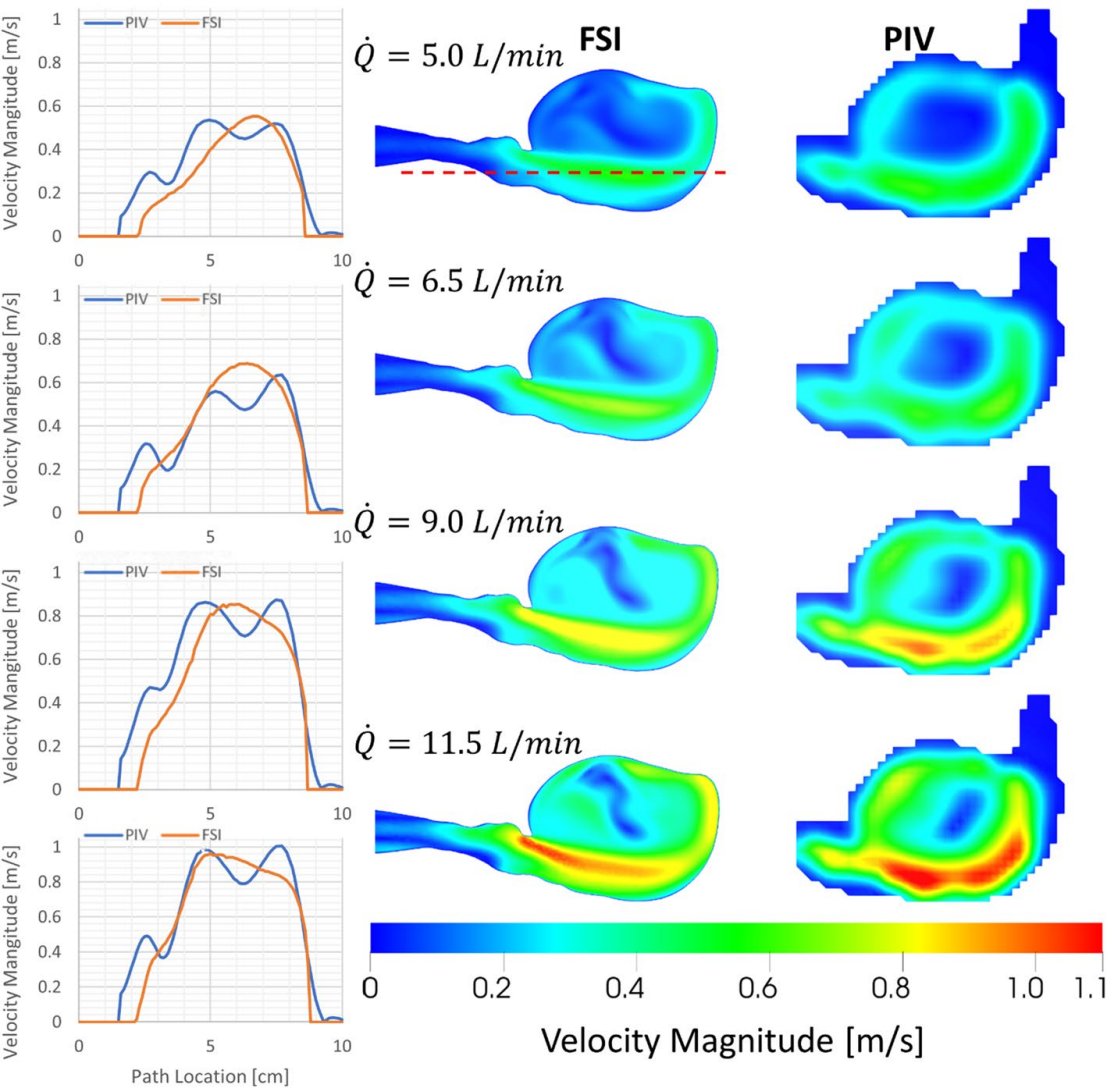
Velocity profile comparison under constant flow rate conditions [1st column: velocity magnitude comparison along path (path is shown with red dashed line in first contour plot under 2nd column), 2nd column: flow velocity from FSI, and 3rd column: flow velocity from PIV]

Flow velocities at the cross-section cut plane (Fig. 7a) and going through the axis of the aorta were compared. Figure 1 provides a visual comparison of flow velocity data in the form of contour plots and line graphs. The path location for the line graph comparison was chosen to cross the sac entering the jet stream, which also has the maximum velocity value. Comparing the experimental flow velocity values from PIV and calculated values from FSI, it was observed that the peak velocity magnitudes were captured accurately at different constant flow rate values. Peak velocity magnitudes (Table 2) show − 7% to + 5% differences at all velocity ranges. The corresponding Pearson correlation coefficient at all 4 flow rates is 0.85, as shown in Table 3.

**Table 2:** Comparison of peak flow velocities calculated by the FSI simulations and measured under constant flow rate conditions

Flow rate (L/min)	Peak V_experimental_ (m/s)	Peak V_FSI_ (m/s)	Relative difference with respect to V_experimental_ (%)
4.9	0.536	0.554	− 3.32
6.7	0.637	0.688	− 7.47
9.1	0.875	0.855	2.29
11.6	1.007	0.963	4.61

**Table 3:** Pearson correlation coefficient (PCC) for constant flow rate conditions

Flow Rate (L/min)	PCC
4.9	0.840
6.7	0.842
9.1	0.844
11.6	0.855

Correlation coefficients were calculated using the velocity magnitude data along the path depicted in Fig. 1

### Pulsatile flow conditions: flow velocity and vectors

**Fig.2:**
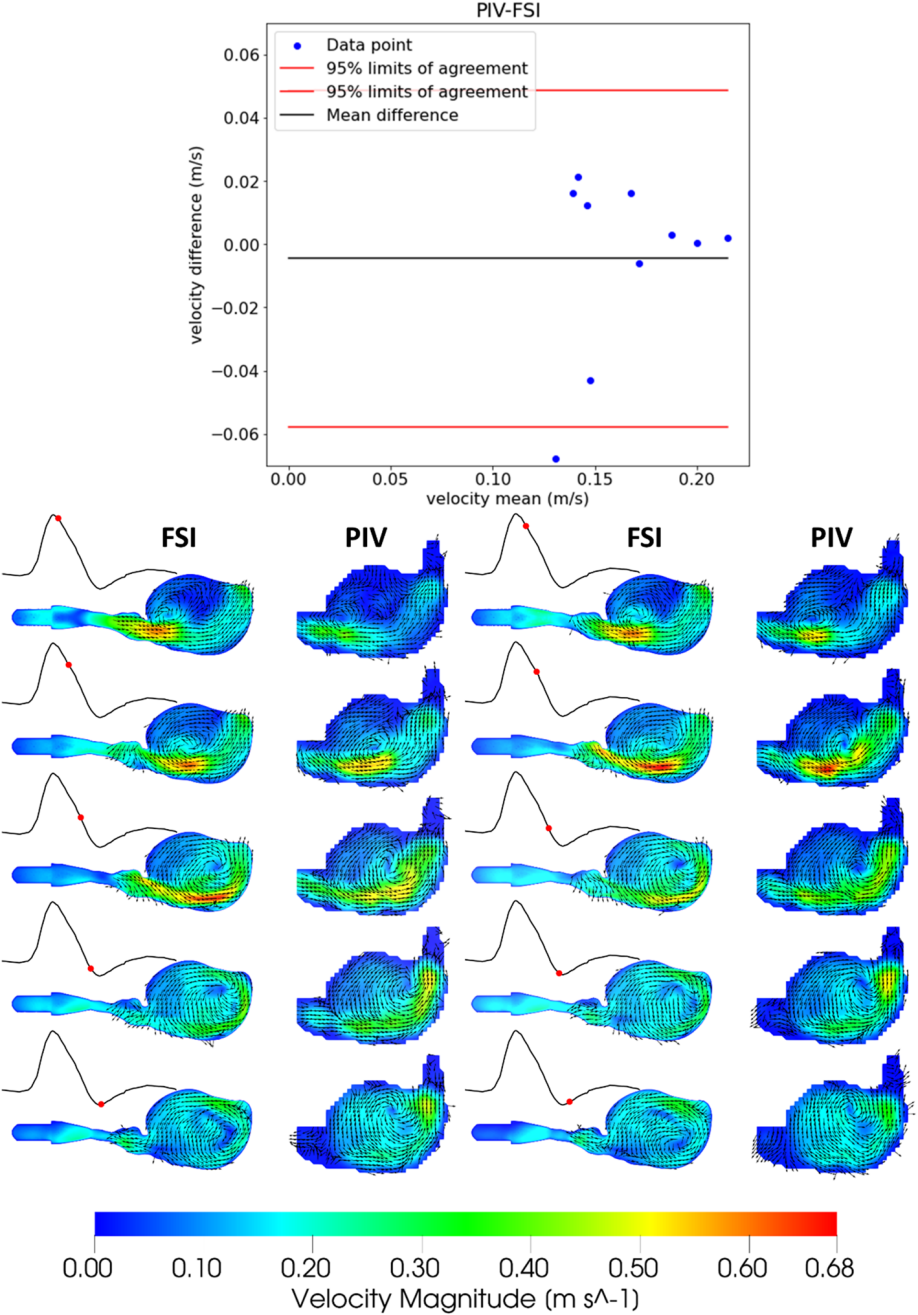
Flow velocities and vectors under pulsatile conditions (60 BPM) (1st and 3rd columns are FSI, and 2nd and 4th columns are PIV data)

Transient pulsatile flow during the cardiac cycle with 60 beats per minute was analyzed next. The aim was to assess the capability of the developed system to capture aneurysm hemodynamics. Both the velocity magnitude and direction using a contour plot with velocity vectors were compared. (Fig. 2) Ten time steps spanning systole and the region after systole, where a high-speed jet coming from upstream enters the aneurysm sac and a vortex ring is formed, were analyzed. This part of the cardiac flow cycle is critical, as it has the highest velocity values, and circulation within the aneurysm sac is encountered. A Bland‒Altman plot based on the cross-section average velocity was generated to show agreement between the PIV and FSI methods.

### Pulsatile flow conditions: vorticity and vortex surface

**Fig. 3:**
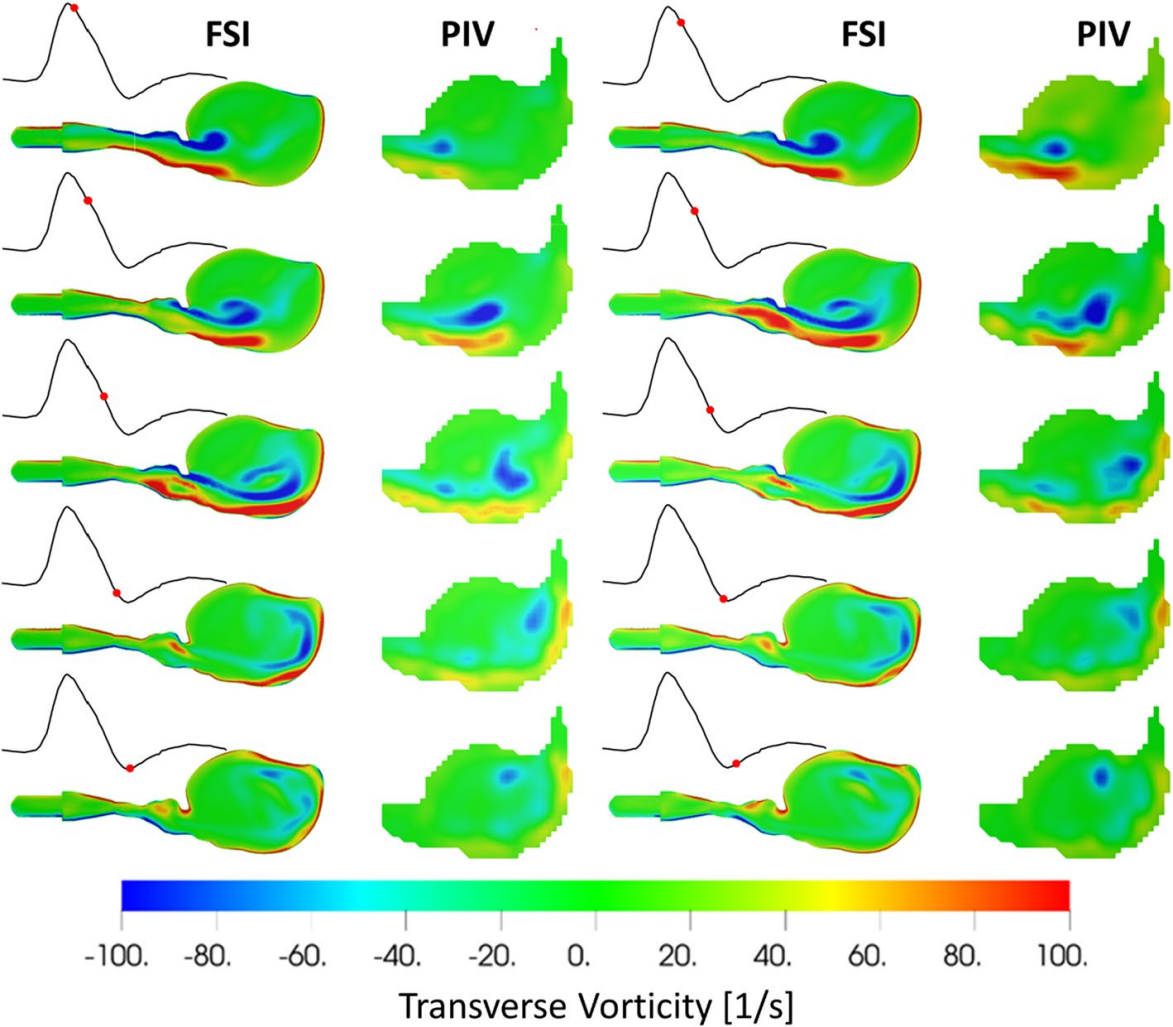
Pulsatile flow transverse vorticity and swirl strength (1st and 3rd columns are FSI, and 2nd and 4th columns are PIV data)

Finally, turbulent structures within the aneurysm sac were analyzed by plotting the transverse vorticity on the same section cut. Figure 3 shows the transverse vorticity contour plot at the same ten time instances spanning the second half of the systole. A comparison of transverse vorticity shows good agreement in terms of the magnitude and location of the low and high vorticity regions between FSI and PIV. Increased cross-section at the aneurysm sac, combined with the accelerating phase of the cardiac cycle, results in the jet entering the sac. The area around this jet wraps into vortices and forms a ring-like structure, as shown by the swirl strength isosurface.

## Discussion

It was shown that comprehensive patient-specific in silico aneurysm hemodynamics can be studied using both numerical and experimental workflow. A patient-specific AAA phantom was fabricated, and hemodynamics were studied under constant and pulsatile flow conditions using an in-house developed affordable PIV setup. The results from each setup were compared against a numerical analysis performed using the FSI method. Despite some large deviations of wall deformations under high constant flow rate conditions, which were over the normal cardiac cycle flow rate range, differences in wall displacements, velocity magnitudes and vectors and turbulent structures were insignificant between PIV and FSI methods. These findings supported the idea that a patient-specific phantom can be manufactured and analyzed experimentally and numerically using an affordable flow circulation simulator and measurement setup.

The flow jet enters the aneurysm sac immediately after the systole peak point and starts moving toward the bottom region. By doing so, it pulls the low-speed flow at the center of the sac with its momentum and creates a downstream moving vortex. The velocity magnitude reaches its peak around the center of the aneurysm sac and starts decreasing afterward toward the end of systole. A side-by-side comparison of the flow velocity magnitudes and vectors (Fig. 2) from FSI and PIV again confirms the correlation between the experimental and numerical systems.

The flow velocity distribution shows a low-velocity region (Fig. 2) at the center of the aneurysm sac, suggesting the presence of a large vortex. The velocity magnitude increases with an increased flow rate and is higher toward the aneurysm surface due to the large vortex. The good match between PIV and FSI again suggests the validity of the integrated workflow.

Deplano [12] and Bauer [14] studied the pulsatile flow over AAA and identified and examined the 3D vortex ring formed during systole. We were able to capture this important phenomenon in our setup as well (Figs. 2 and 3).

We note that the pump used in the flow circulation can be improved with a noncorroding gear and casing material or a no-contact piston type pump instead of a gear pump. This will decrease blurring and improve PIV robustness.

This study used 2D PIV, as it does not require an extra synchronization setup. The global shutter camera used in this study can also be used for 2.5D stereo PIV and 3D particle image tracking (PTV). There are sync and trigger capabilities on the camera board. We plan to increase the number of cameras in the system to enable higher dimensional measurements.

Geoghegan et al. used LED illumination in place of a double-pulsed laser system as a low-cost alternative [19]. We have considered this, but were not able to implement within the scope of this current work. The use of LED illumination via fiber optics stands out as a further alternative cost reduction item for analysis.

Our experiments were limited by controlled flow conditions. The inlet flow rate was numerically controlled, but the outlet pressure and temperature of the blood phantom were not controlled. We understand that a temperature control unit and Windkessel model elements downstream are required to better capture physiological conditions. However, the goal of this study was constrained to show that a low-cost patient-specific phantom can be manufactured and analyzed for hemodynamics using an in-house built PIV system. Some researchers [31, 38, 54] opted to use physiological outlet pressure values, the three-element Windkessel model [32, 42, 55] or calculated loss coefficients [41], whereas others [34] used zero-pressure conditions. All remaining walls were set as no-slip wall conditions. Since the main aim of the simulations was to validate PIV measurements, boundary conditions were chosen to match experimental conditions.

Anisotropic wall properties, intraluminal thrombus (ILT), and calcifications all alter wall stress levels and thus are important in the assessment of AAA wall rupture risk [24]. We did use linear elastic material properties following Yazdi et al. [21] and constant wall thickness. We observed high discrepancies under increased flow rates (see Table 1), which suggests that a non-linear material model may be more appropriate. We plan to use more advanced hyperelastic material models and perform material calibration using image-based measurements as [36].

There has been a recent study [20] on using anisotropic arterial walls by multimaterial 3D printing. Another study [18] outlined a tunable wall compliance system for the aorta. We understand that the presented workflow can be improved by considering patient-specific wall properties and variable thickness by the above-mentioned methods, but PIV method would not be compatible as it relies on clear view through PDMS walls.

Finally, a reference particle image velocimetry system was not available. Rotating disk experiment was employed instead for validating the low-cost particle image velocimetry system measurements on AAA hemodynamics. Despite all limitations, we were able to show that a patient-specific vascular phantom can be manufactured using 3D printing and that hemodynamics can be analyzed again using an in-house PIV system and supported by numerical models.

The key pillars enabling low-cost patient-specific in vitro experiments can be listed as open-source software (itkSnap) used in medical image processing and PIV analysis (pivLab), affordable ($100) global shutter cameras, and FDM-type 3D printers ($650). We were able to show that combining these technologies can help democratize vascular in vitro research.

## Conclusions

With lowered costs of experimentation and validation capabilities, we envision more researchers to set up wet experiments and study more pathologies and test vascular devices under realistic flow and topological conditions. Numerical studies that lack experimental validation will have easy access to experimental validation.

The presented workflow and techniques are expected to lower the barrier for endovascular research and device development testing. The complete workflow presented in this paper can also be extended to other aortic aneurysms as well as cerebral aneurysms. With more testing and validation, broader populations can obtain access to minimally invasive endovascular treatment with decreased device-related mechanical failure risk.

## Methods

### Patient-specific flexible phantom manufacturing

A thin-walled, compliant design was chosen to accurately [40] capture AAA hemodynamics and interactions with flexible synthetic systems. Phantom was manufactured using a lost-core casting [16] technique, where molds were generated via a consumer grade FDM 3D printer (Prusa MKS).

**Fig. 4:**
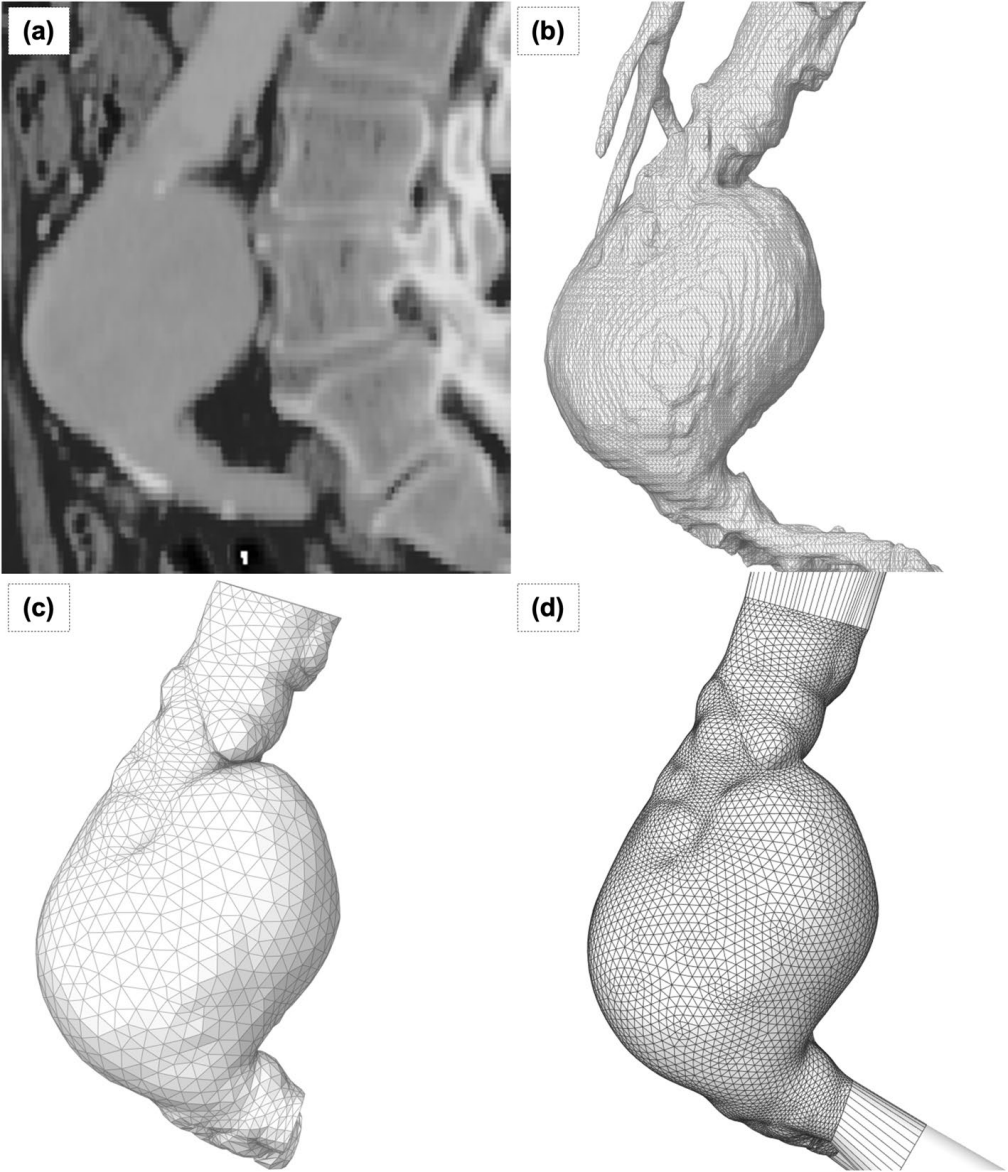
Patient-specific medical image processing for modeling. **a** Panaromix DICOM Image, **b** STL Surface (Output from ITK-Snap), **c** Smooth, Decimate and Expand 2 mm Wall Thickness, and **d** Final Smooth, Remesh for Mold Design, CFD, and FEA

Medical images (Fig. 4a) from an abdominal CT angiogram of a patient with AAA (alias name = PANORAMIX)) were taken from the Osirix DICOM image library [56]. The 3D surface model of the AAA (Fig. 4b) was extracted from the medical image data set using the region competition snakes method, available in ITK-SNAP [57] software.

The CAD model for the casting mold was generated using Ansys Space Claim [58]. The standard tessellation language (STL) geometry obtained from segmentation was processed to filter out noise in the surface data and fix errors. The surface model was extruded in local normal directions at a constant wall thickness of 2 mm to obtain a 3D AAA wall representation (Fig. 4c). The NURBS surface fit operation on the STL geometry was then performed to allow Boolean operations needed for mold design (Fig. 4d).

**Fig. 5:**
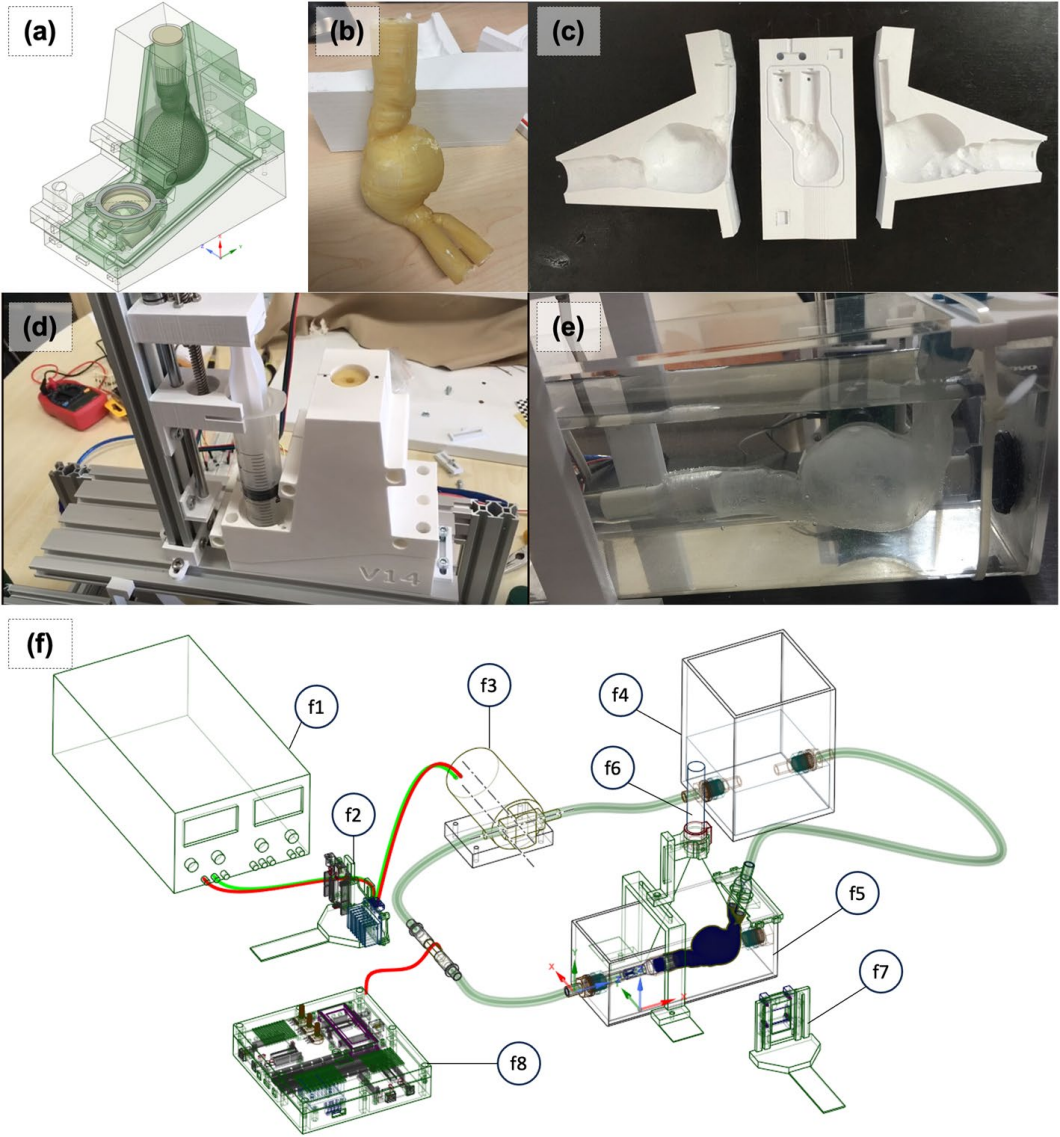
Lost-core Casting Mold Design, Phantom Manufacturing and Flow Circuit Setup. **a** Mold design for 3D printing. **b** Male mold 3D printed using PVA. **c** Female mold 3D printed using PLA. **d** Injection process of Sylgard184 © Flexible phantom inside blood-mimicking fluid. **f** Flow circuit setup with power source (f1), motor drive (f2), gear pump (f3), reservoir (f4), phantom pool (f5), laser (f6), image sensor (f7), and control and measurement cards (f8)

For lost-core casting, a set of molds was designed (Fig. 5a) and manufactured in pieces. The male mold (Fig. 5b), which defines the arterial lumen, was printed using a water-soluble polyvinyl alcohol (PVA) filament due to the complex shape of the aneurysm geometry. The female mold, which defines the outer surfaces of the aorta geometry, is sliced into three pieces (Fig. 5c) to allow proper opening of the mold after curing.

A layer thickness of 0.1 mm was chosen, because it provided a good balance between accuracy, smoothness, and post-processing needed. Mold surfaces were postprocessed using abrasive paper of different grits in the range of 240 to 5000.

Polydimethylsiloxane (PDMS) (commercial name Slygard 184) was used as the cast material. Optical errors in PIV measurement were minimized by matching refractive indices of the arterial wall and blood-mimicking fluid at 1.41. The PDMS mixture was degassed using an in-house vacuum chamber to prevent bubble formation. Casting of PDMS was accomplished by an in-house built mold rotation plus syringe control mechanism (Fig. 5d). Coordinating the mold orientation with the injection volume was required to prevent air entrapment within the mold. Curing was completed at room temperature over 48 h. After curing, the female mold pieces were separated, and the PVA male mold was allowed to dissolve in warm water.

With a flexible, optically clear, patient-specific phantom (Fig. 5e) manufactured, the next step was to connect the phantom to a flow circuit simulating pulsatile flow conditions and perform PIV measurements for hemodynamics studies.

### Flow circuit and image acquisition for PIV

**Fig. 6:**
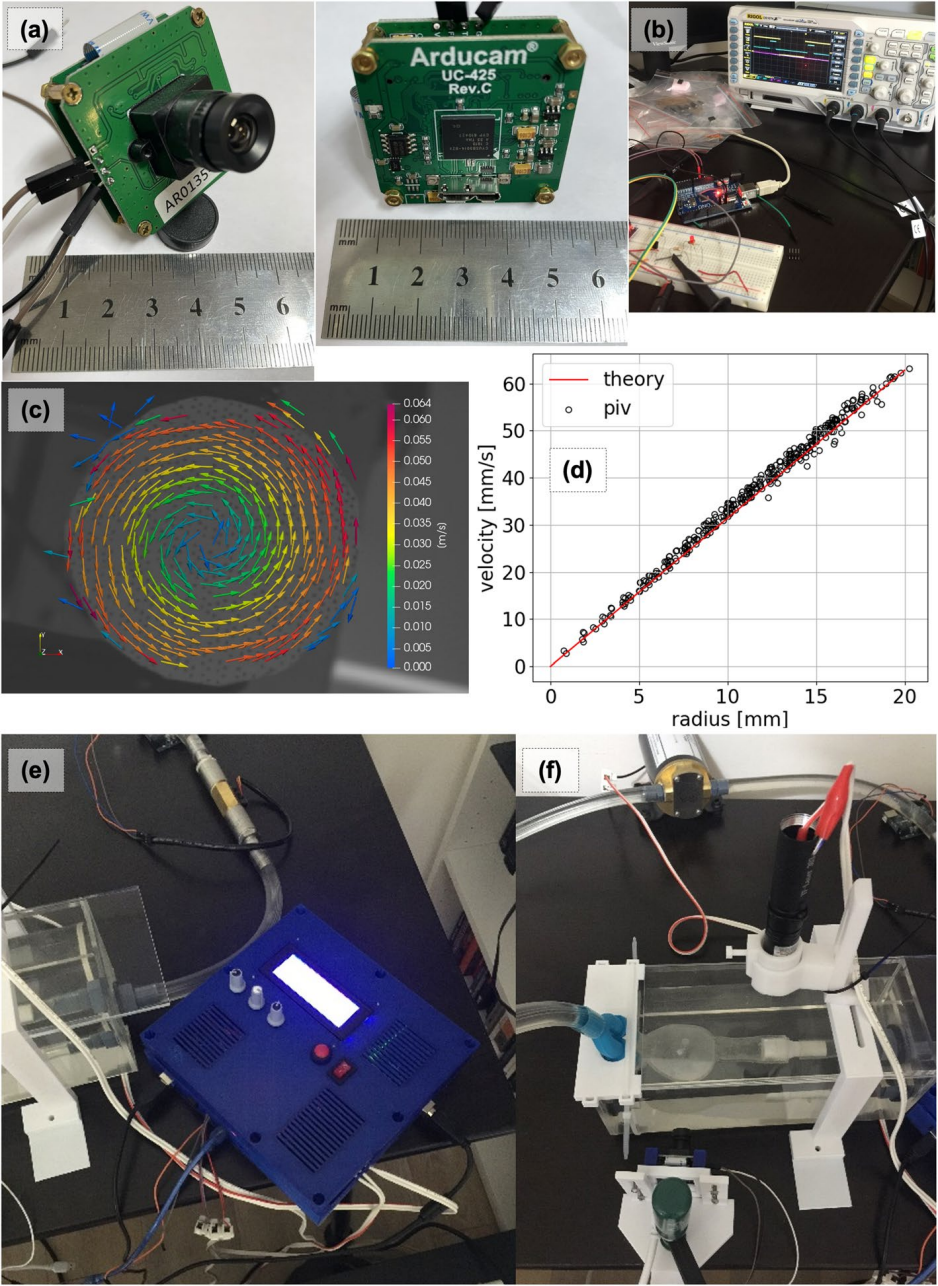
Particle image velocimetry setup. **a** Global shutter camera. **b** Camera and laser synchronization. **c** PIV validation with rotating disk. **d** Velocity over radius on Di©. **e** Control box and flow meter. **f** Laser and camera positioning

A flow circuit (Fig. 5f) was built in-house using off-the-shelf components. A gear pump (Fig. 6f) with a 24 V DC motor (SeaFlo 3.2GPM) was numerically controlled using a motor driver and Arduino controller (Fig. 6d). The input flow rate versus timetable, replicating the pulsatile blood flow, was discretized to 8 bit pulse levels. The gear pump was calibrated separately using a volumetric flow meter (Fig. 6f). The phantom was connected to a flow circuit and assembled inside an acrylic plexiglass pool filled with a blood-mimicking liquid (Fig. 5e) to achieve zero light refraction. An extra pool was used to balance blood-mimicking fluid in the system and easy removal of bubbles within the fluid. A synchronized laser and camera system (Fig. 6e) was attached around the phantom for PIV measurements.

Blood-mimicking liquid was prepared using a water (48%), glycerol (37%), and NaCl (15%) mixture, in line with Gulan et al. [59]. Tracking particles were also manufactured in-house using the techniques presented by Pedocchi et al. [60]. Rhodamine-added polyester was cured and crushed. A sieve system was used to obtain particle sizes between 60 µm and 100 µm. Particles were added into the blood-mimicking liquid as required to achieve PIV-needed particle density of 10 particles per interrogation window.

A low-cost image acquisition system was built to capture image data needed for PIV analysis. Based on the expected peak flow velocity of ~ 1.0 m/s in the aneurysm sac and the region of interest, it was concluded that a global shutter camera was needed. The AR0135 mono image sensor (Fig. 6a) from ONSEMI was used, because it allowed timing control through registry, trigger, and flash signal output at an affordable price of ~ $100. AR0135 provides a 60 frames per second capture rate at 1280 × 960 pixel resolution. A double laser pulse, generated by a 1000 mW, 532 nm wavelength green laser pointer model LT301(Fig. 6e), was synchronized with imaging cycles of the global shutter camera as needed by the frame straddling technique. For synchronization, the FLASH channel of the AR0135 image sensor was listened to with an Arduino, and the laser was triggered twice with a 400 µs offset using custom Arduino code. A cylindrical lens was used to expand the laser beam into a light sheet. The internal circuit of the laser pointer was not modified, but the light generation characteristics were validated using a light sensor connected to an oscilloscope (Fig. 6b). Laser input signals were adjusted to align actual light intensity with camera exposure.

A PIV validation experiment was set up using a rotating disk with reflective particles (Fig. 6c) as in Cierpka et al. [52]. The speed of the motor was independently monitored using a laser speedometer and set to 30 RPM. Image sets were then analyzed in the PIV software to compare the rotational speed of the disk obtained from PIV with laser speedometer readings. The scatter plot of radius vs velocity in Fig. 6d shows a good fit (standard deviation: 0.27 mm/s) between the theoretical speed and PIV.

PIVLab software [61] was used to calculate velocity vector fields from PIV image pairs. An interrogation window of 32 × 32 pixels with 16px overlap was chosen. The PIV method employed was double-frame PIV due to a limiting frame rate of 60 for the AR0135 image sensor. Calculated velocity vectors were written in VTK format to be compared against results obtained from numerical simulations.

With the experimental setup ready, the final step of the study was to build an identical numerical model of the phantom and the flow conditions to validate measurements.

### Fluid–structure interaction analysis

A fluid–structure interaction model was built to validate the PIV measurements performed on the patient-specific, flexible AAA phantom. Both steady-state and transient FSI analyses were performed, and the velocity/vorticity results were compared against the PIV measurement data.

**Fig. 7:**
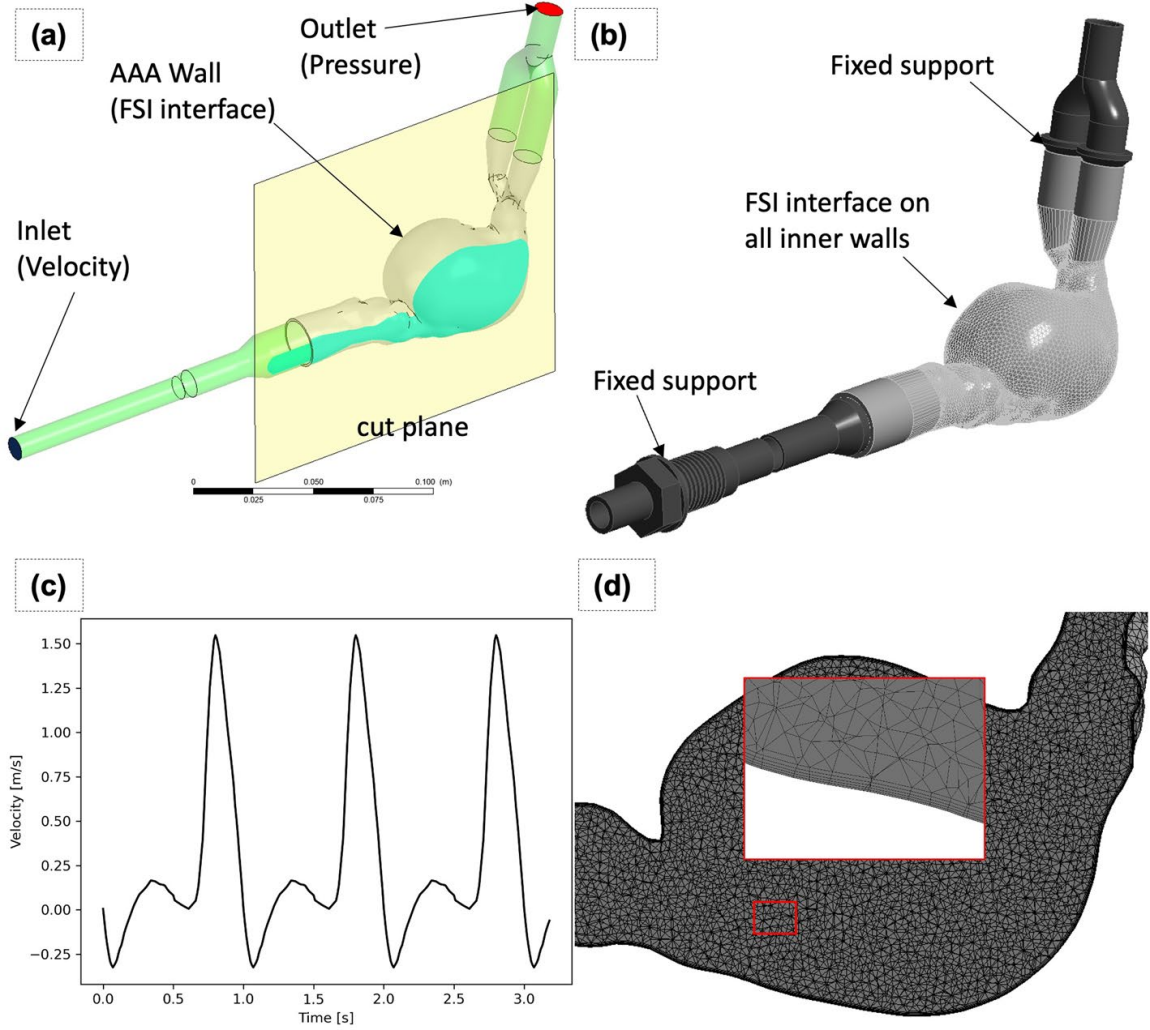
Fluid–structure interaction setup, mesh and boundary conditions. **a** The CFD domain has 1 inlet with a time varying velocity profile and 1 zero-pressure outlet. The yellow cut plane used for PIV is located at Y = 8 mm. **b** The FEA domain has two fixed supports upstream and downstream, and all internal walls are assigned as interfaces to the CFD model. **c** Inlet flow rate as a function of time, based on Deplano et al. **d** CFD mesh and boundary layer mesh detail

The initial fluid domain for the blood-mimicking fluid (Fig. 7a) was generated by cavity filling operations in CAD software. The solid domain of the phantom for the structural FE model was imported from the mold design phase. Additional hose and plastic connectors were modeled and assembled (Fig. 7b) to the phantom model. Both solid and fluid domains were meshed using Ansys Workbench software [58]. Boundary-layer inflation meshing (Fig. 7d) was used on all walls, with an initial layer thickness of 75 µm. The correctness of the initial layer thickness was confirmed by y-plus values of approximately 1. The CFD mesh consisted of 172 K nodes and 665 K elements. Structural mesh was also generated in Ansys Workbench meshing using high-order elements of SOLID186 type. Element sizing was specified as 1.25 mm, and the minimum element count through arterial wall thickness was set to 2 [38]. The structural mesh had 546 K nodes and 303 K elements. A mesh size of 1.25 mm was used for the CFD domain, based on Les et al. [32]. A detailed mesh convergence study was performed per [62, 63] to confirm the selected mesh parameters. Differences in measured quantities, namely displacement and velocity, were found out to be less than 1% and 4%, respectively, between the chosen mesh and the finer mesh (Additional file 1: Table S1–S3 and Fig. S1–S7).

Ansys [58] Mechanical and CFX were used to perform FSI analysis based on a pressure–displacement transfer weak coupling. The coupling process needed fine tuning of the default settings, as defaults resulted in solution instability. First, the wall forces calculated in the CFD solver were damped within the CFX solver by specifying an internal relaxation factor per inner CFD loop iteration. On the system coupling level, the forces calculated in the CFD solver were transferred to the mechanical solver using a 0.5 relaxation factor. A minimum of ten coupling iterations was enforced to ensure accurate calculation of force levels. All coupling iterations were confirmed to converge according to the 1e−2 force and 1e−3 displacement criteria, within maximum 20 iterations. CFD RMS residual target was set as 1e−4. All solver setup details are listed in Additional file 1: Table S4.

Blood-mimicking fluid viscosity was calculated as 5.82e−3 Pa s using reported kinematic viscosity of 4.85e−6 m^2^/s and density of 1200 kg/m^3^ in Gulan et al.’s study [59].

Linear elastic material models were used for all parts in the structural model. The mechanical properties of the structural parts, including the PETG plastic connectors [64], are listed in Table 4.

**Table 4:** Structural properties for FEA simulation

Material	Mechanical properties
PETG	Density: 1230 kg/m^3^ Material model: Linear elastic Young’s modulus: 1.17 GPa Poisson’s ratio: 0.35
PDMS (SYLGARD)	Density: 1000 kg/m^3^ Material model: Linear elastic Young’s modulus: 0.705 MPa Poisson’s ratio: 0.4

**Fig. 8:**
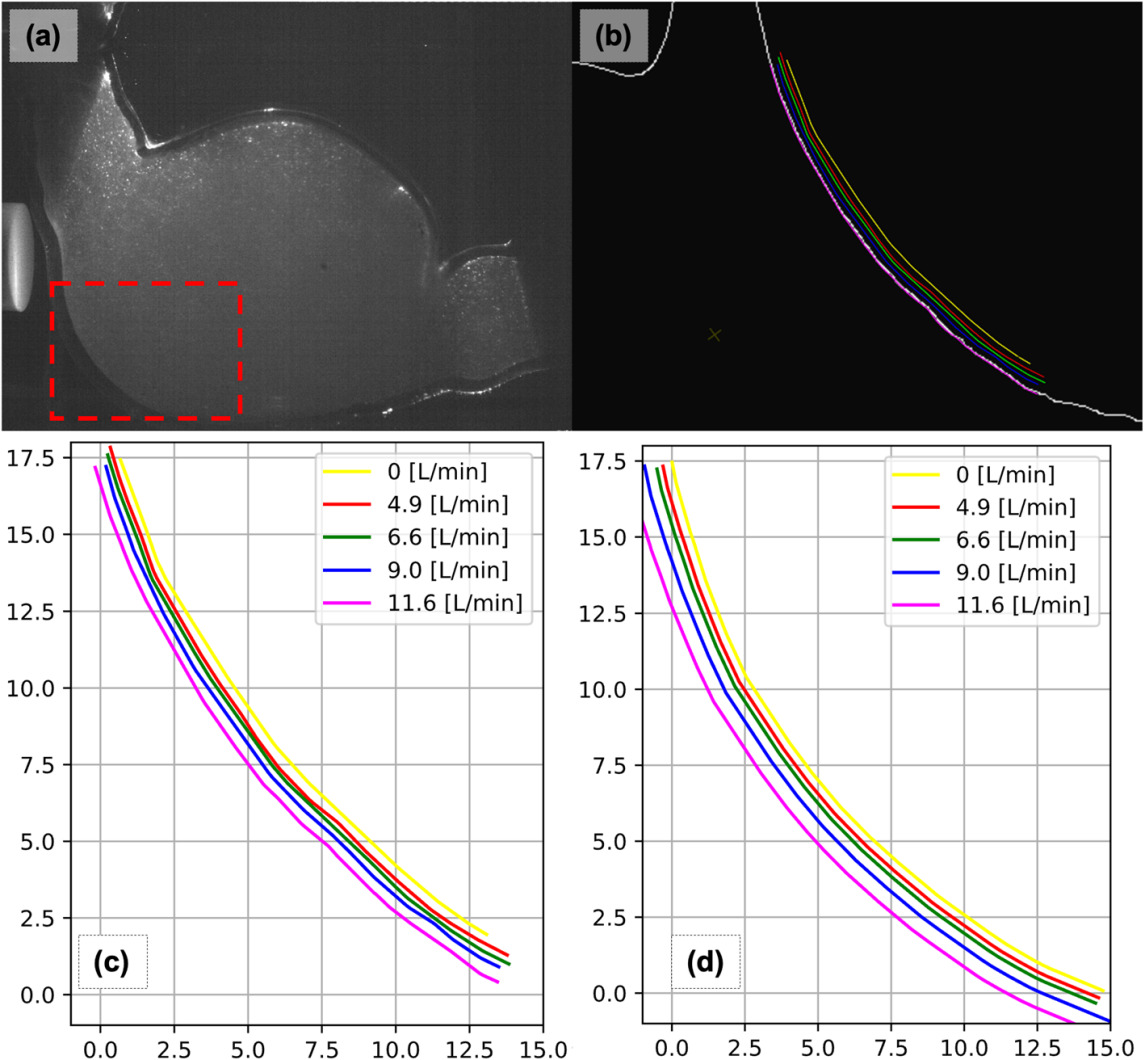
Constant flow wall deformation and calibration: **a** PIV raw image, **b** wall edge extraction, **c** experimentally measured wall deformations, and (d) FSI calculated wall deformations

PDMS stiffness was tuned, such that wall displacements calculated using steady-state FSI simulations match experimental measurements under constant flow conditions. The same cut plane (Fig. 7a) used for FSI and PIV analysis was used. Experimental wall deformations were extracted (Fig. 8a) from PIV images using an edge detection (Fig. 8b) image processing algorithm from the OpenCV Python library. The displacements between each displaced wall curve were measured in CAD, again using a custom Python script in Ansys SpaceClaim.

The SST k-omega turbulence model of CFX was used, based on the literature [37] for analysis of aort and aort aneurysm hemodynamic simulations. Tan et al. found that the SST model correlates well with MR data [34, 45].

For pulsatile flow analysis, a time step size of 0.01 s was used. Tan et al. performed a convergence study on time step size and found that the difference in velocity and turbulence kinetic energy values are within 1% for time step sizes from 0.01 s to 0.0025 s [34]. Comparison of temporal values showed no meaningful difference based on time step sizing, suggesting 0.01 s to be a suitable time step size for analysis.

The structural model was assumed to be stationary at the beginning of the analysis. The starting time instances correspond to the end of systole. Similarly, both the velocity and pressure fields of the fluid flow solution were initialized with zero values.

The upstream and downstream ends of the structural domain were assigned fixed boundary conditions (Fig. 8b), where all three displacement degrees of freedom were fixed. The inner arterial walls were set as coupling boundary conditions where the pressure field from the fluid flow solution was interpolated onto the structural model.

For pulsatile flow simulations, the inlet flow rate was specified at the proximal end of the aorta using the flow rate vs time profile (Fig. 8c) from Deplano et al.’s study [12]. The outlet boundary (Fig. 8a) was set to be a relative zero-pressure condition.

All simulations were run in parallel using Ansys built-in parallel solvers. A Windows Server 2016 with Intel Xeon processor was used. Both the finite-element analysis and CFD parts of the solution were solved using eight cores. The solution time per time step was approximately 50 min, which corresponds to approximately 10 days for 3 cardiac cycles at 60 beats per minute.

Paraview was employed to postprocess the PIV and FSI analysis results. The swirl strength was not readily available at the time of this paper, so we developed a Python script plugin based on Chakraborty et al. [65].

Pearson’s correlation coefficient (PCC) was calculated to compare strength of the linear relationship of velocity values between PIV and FSI analysis for the constant flow rate cases. Bland–Altman plots were generated to assess the difference between PIV and FSI velocity values.

### Supplementary Information

**Additional file 1:** **Table S1.** Grid parameters and the total number of the CFD and mechanical domains.** Table S2.** Mesh sensitivity results for constant flow at 11.5 L/min flow rate condition. **Table S3.** Mesh sensitivity results for pulsatile flow at 60 BPM condition. **Table S4.** Solver parameters for the transient FSI run. **Figure S1.** Total wall displacement under constant flow (11.5 L/min flowrate) condition (Coarse-Chosen-Fine Mesh). **Figure S2.** Velocity at cut plane under constant flow (11.5 L/min flow rate) condition. **a** Fine, **b** chosen, **c** coarse mesh. **Figure S3.** Comparison of maximum velocity of the domain vs time, pulsatile flow at 60 BPM condition. **Figure S4.** Comparison of space averaged velocity over time for cut-plane, pulsatile flow at 60 BPM condition. **Figure S5.** Time average wall shear stress (TAWSS), pulsatile flow at 60 BPM condition. **Figure S6.** Time averaged displacement (TAD), pulsatile flow at 60 BPM condition. **Figure S7.** Space averaged Von-Mises stress at wall, pulsatile flow at 60 BPM condition.

## Data Availability

The datasets generated and/or analyzed during the current study are available in the Zenodo data base, with DOI number 10.5281/zenodo.10068458.

## Abbreviations

AAA: Abdominal aorta aneurysms
EVAR: Endovascular aneurysm repair
PIV: Particle image velocimetry
FSI: Fluid–structure interaction
MRI: Magnetic resonance imaging
CFD: Computational fluid dynamics
PCC: Pearson correlation coefficient
PTV: Particle image tracking
PDMS: Polydimethylsiloxane
ILT: Intraluminal thrombus

## References

[1] Adam van der Vliet J, Boll AP (1997). Abdominal aortic aneurysm. Lancet.

[2] Choke E, Cockerill G, Wilson WRW, Sayed S, Dawson J, Loftus I (2005). A review of biological factors implicated in abdominal aortic aneurysm rupture. Eur J Vasc Endovasc Surg.

[3] Vorp DA (2007). Biomechanics of abdominal aortic aneurysm. J Biomech.

[4] Brewster DC, Cronenwett JL, Hallett JW, Johnston KW, Krupski WC, Matsumura JS (2003). Guidelines for the treatment of AAAs. J Vasc Surg.

[5] Gasser TC, Nchimi A, Swedenborg J, Roy J, Sakalihasan N, Böckler D (2014). A novel strategy to translate the biomechanical rupture risk of abdominal aortic aneurysms to their equivalent diameter risk: method and retrospective validation. Eur J Vasc Endovasc Surg.

[6] Uberoi R, Jenkins M (2020). Is this the end for EVAR?. Cardiovasc Interv Radiol.

[7] Li Z, Kleinstreuer C (2005). Blood flow and structure interactions in a stented abdominal aortic aneurysm model. Med Eng Phys.

[8] Li Z, Kleinstreuer C (2006). Analysis of biomechanical factors affecting stent-graft migration in an abdominal aortic aneurysm model. J Biomech.

[9] Kleinstreuer C, Li Z, Farber MA (2007). Fluid-structure interaction analyses of stented abdominal aortic aneurysms. Annu Rev Biomed Eng.

[10] Deplano V, Knapp Y, Bertrand E, Gaillard E (2007). Flow behaviour in an asymmetric compliant experimental model for AAA. J Biomech.

[11] Deplano V, Meyer C, Guivier-Curien C, Bertrand E (2013). New insights into the understanding of flow dynamics in an in vitro model for AAAs. Med Eng Phys.

[12] Deplano V, Guivier-Curien C, Bertrand E (2016). 3D analysis of vortical structures in an AAA by stereoscopic PIV. Exp Fluids.

[13] Wang Y, Joannic D, Patrick J, Keromnes A, Aur M, Lalande A (2017). Comparison of flow measurement by 4D flow magnetic resonance imaging and by particles image velocimetry on phantom of abdominal aortic aneurysm. SM Vasc Med.

[14] Bauer A, Bopp M, Jakirlic S, Tropea C, Krafft AJ, Shokina N (2020). Analysis of the wall shear stress in a generic aneurysm under pulsating and transitional flow conditions. Exp Fluids.

[15] Boersen JT (2017). Validation of endovascular aneurysm sealing for treatment of abdominal aortic aneurysm. Ecosyst Serv.

[16] Annio G, Franzetti G, Bonfanti M, Gallarello A, Palombi A, De Momi E (2020). Low-cost fabrication of polyvinyl alcohol-based personalized vascular phantoms for in vitro hemodynamic studies: three applications. J Eng Sci Med Diagn Ther.

[17] Chi Q-Z, Mu L-Z, He Y, Luan Y, Jing Y-C (2021). A brush–spin–coating method for fabricating in vitro patient-specific vascular models by coupling 3D-printing. Cardiovasc Eng Technol.

[18] Gallarello A, Palombi A, Annio G, Homer-Vanniasinkam S, De Momi E, Maritati G (2019). Patient-specific aortic phantom with tunable compliance. J Eng Sci Med Diagn Ther.

[19] Geoghegan PH, Buchmann NA, Soria J, Jermy MC (2013). Time-resolved PIV measurements of the flow field in a stenosed, compliant arterial model. Exp Fluids.

[20] Ruiz de Galarreta S, Antón R, Cazón A, Finol EA (2017). A methodology for developing anisotropic AAA phantoms via additive manufacturing. J Biomech.

[21] Yazdi SG, Huetter L, Docherty PD, Williamson PN, Clucas D, Jermy M (2019). A novel fabrication method for compliant silicone phantoms of arterial geometry for use in particle image velocimetry of haemodynamics. Appl Sci Switz..

[22] Geoghegan PH, Buchmann NA, Spence CJT, Moore S, Jermy M (2012). Fabrication of rigid and flexible refractive-index-matched flow phantoms for flow visualisation and optical flow measurements. Exp Fluids.

[23] Yazdi SG, Geoghegan PH, Docherty PD, Jermy M, Khanafer A (2018). A review of arterial phantom fabrication methods for flow measurement using PIV techniques. Ann Biomed Eng.

[24] Xenos M, Bluestein D (2011). Biomechanical aspects of abdominal aortic aneurysm (AAA) and its risk of rupture : fluid structure interaction (FSI) studies. Small.

[25] Reymond P, Crosetto P, Deparis S, Quarteroni A, Stergiopulos N (2013). Physiological simulation of blood flow in the aorta: comparison of hemodynamic indices as predicted by 3-D FSI, 3-D rigid wall and 1-D models. Med Eng Phys.

[26] Mendez V, Di Giuseppe M, Pasta S (2018). Comparison of hemodynamic and structural indices of ascending thoracic aortic aneurysm as predicted by 2-way FSI, CFD rigid wall simulation and patient-specific displacement-based FEA. Comput Biol Med.

[27] Drewe CJ, Parker LP, Kelsey LJ, Norman PE, Powell JT, Doyle BJ (2017). Haemodynamics and stresses in abdominal aortic aneurysms: a FSI study into the effect of proximal neck and iliac bifurcation angle. J Biomech.

[28] Qiao Y, Luo K, Fan J (2022). Component quantification of aortic blood flow energy loss using computational fluid–structure interaction hemodynamics. Comput Methods Progr Biomed.

[29] Qiao Y, Fan J, Luo K (2023). Mechanism of blood flow energy loss in real healthy aorta using computational fluid–structure interaction framework. Int J Eng Sci.

[30] Chen CY, Anẗon R, Hung MY, Menon P, Finol EA, Pekkan K (2014). Effects of intraluminal thrombus on patient-specific AAA hemodynamics Via stereoscopic PIV and CFD modeling. J Biomech Eng.

[31] Kung EO, Les AS, Medina F, Wicker RB, McConnell MV, Taylor CA (2011). In vitro validation of finite-element model of aaa hemodynamics incorporating realistic outlet boundary conditions. J Biomech Eng.

[32] Les AS, Shadden SC, Figueroa CA, Park JM, Tedesco MM, Herfkens RJ (2010). Quantification of hemodynamics in abdominal aortic aneurysms during rest and exercise using magnetic resonance imaging and computational fluid dynamics. Ann Biomed Eng.

[33] Steinlauf S, Hazan Shenberger S, Halak M, Liberzon A, Avrahami I (2021). Aortic arch aneurysm repair: unsteady hemodynamics and perfusion at different heart rates. J Biomech.

[34] Tan FPP, Borghi A, Mohiaddin RH, Wood NB, Thom S, Xu XY (2009). Analysis of flow patterns in a patient-specific thoracic aortic aneurysm model. Comput Struct.

[35] Antón R, Chen CY, Hung MY, Finol EA, Pekkan K (2015). Experimental and computational investigation of the patient-specific abdominal aortic aneurysm pressure field. Comput Methods Biomech Biomed Engin.

[36] Meyer CA, Bertrand E, Boiron O, Deplano VV (2011). Stereoscopically observed deformations of a compliant abdominal aortic aneurysm model. J Biomech Eng.

[37] Alimohammadi M, Sherwood JM, Karimpour M, Agu O, Balabani S, Díaz-Zuccarini V (2015). Aortic dissection simulation models for clinical support: Fluid–structure interaction vs. rigid wall models. Biomed Eng Online.

[38] Molony DS, Callanan A, Kavanagh EG, Walsh MT, McGloughlin TM (2009). Fluid–structure interaction of a patient-specific abdominal aortic aneurysm treated with an endovascular stent-graft. Biomed Eng OnLine.

[39] Ong CW, Kabinejadian F, Xiong F, Wong YR, Toma M, Nguyen YN (2019). Pulsatile flow investigation in development of thoracic aortic aneurysm: an in-vitro validated fluid–structure interaction analysis. J Appl Fluid Mech.

[40] Hirschhorn M, Tchantchaleishvili V, Stevens R, Rossano J, Throckmorton A (2020). Fluid–structure interaction modeling in cardiovascular medicine: a systematic review 2017–2019. Med Eng Phys.

[41] Benim AC, Nahavandi A, Assmann A, Schubert D, Feindt P, Suh SH (2011). Simulation of blood flow in human aorta with emphasis on outlet boundary conditions. Appl Math Model.

[42] Arzani A, Suh GY, Dalman RL, Shadden SC (2014). A longitudinal comparison of hemodynamics and intraluminal thrombus deposition in abdominal aortic aneurysms. Am J Physiol Heart Circ Physiol.

[43] Aycock KI, Hariharan P, Craven BA (2017). Particle image velocimetry measurements in an anatomical vascular model fabricated using inkjet 3D printing. Exp Fluids.

[44] Bordones AD, Leroux M, Kheyfets VO, Wu Y-AA, Chen C-YY, Finol EA (2018). Computational fluid dynamics modeling of the human pulmonary arteries with experimental validation. Ann Biomed Eng.

[45] Cheng Z, Tan FPP, Riga CV, Bicknell CD, Hamady MS, Gibbs RGJ (2010). Analysis of flow patterns in a patient-specific aortic dissection model. J Biomech Eng.

[46] Sugiu KS, Artin JM, Ean BJ, Ailloud PG, Andai SM, Ufenacht DAR (2003). Artificial cerebral aneurysm model for medical testing, training, and research. Neurol Med Chir Tokyo..

[47] Ho CK, Chee AJY, Yiu BYS, Tsang ACO, Chow KW, Yu ACH (2017). Wall-less flow phantoms with tortuous vascular geometries: design principles and a patient-specific model fabrication example. IEEE Trans Ultrason Ferroelectr Freq Control.

[48] Medero R, García-Rodríguez S, François CJ, Roldán-Alzate A (2017). Patient-specific in vitro models for hemodynamic analysis of congenital heart disease: additive manufacturing approach. J Biomech.

[49] Yip R, Mongrain R, Ranga A, Brunette J, Cartier R. Development of Anatomically Correct Mock-Ups of the Aorta for PIV Investigations. Inaug CDEN Des Conf. Montreal, Canada; 2004. p. 1–10.

[50] Souza A, Souza MS, Pinho D, Agujetas R, Ferrera C, Lima R (2020). 3D manufacturing of intracranial aneurysm biomodels for flow visualizations: low cost fabrication processes. Mech Res Commun.

[51] Updegrove A, Wilson NM, Merkow J, Lan H, Marsden AL, Shadden SC (2016). SimVascular: an open source pipeline for cardiovascular simulation. Ann Biomed Eng.

[52] Cierpka C, Otto H, Poll C, Hüther J, Jeschke S, Mäder P (2021). SmartPIV: flow velocity estimates by smartphones for education and field studies. Exp Fluids.

[53] Kashyap V, Kumar S, Jajal NA, Mathur M, Singh RK (2020). Parametric analysis of smartphone camera for a low cost PIV system. arXiv..

[54] Khanafer K, Berguer R (2009). Fluid–structure interaction analysis of turbulent pulsatile flow within a layered aortic wall as related to aortic dissection. J Biomech.

[55] Suh G-Y. Hemodynamic changes quantified in abdominal aortic aneurysms with increasing exercise intensity using magnetic resonance imaging and computational fluid dynamics. PhD Thesis. Stanford University; 2011. 10.1007/s10439-011-0313-6 PMC3362397 21509633

[56] OsiriX DICOM Image Library. OsiriX. https://www.osirix-viewer.com/resources/dicom-image-library/. Accessed 20 Feb 2018.

[57] Yushkevich PA, Piven J, Cody Hazlett H, Gimpel Smith R, Ho S, Gee JC (2006). User-guided 3D active contour segmentation of anatomical structures. Neuroimage.

[58] Ansys User Guide. 2022. http://www.ansys.com.

[59] Gülan U, Kinzelbach W, Holzner M (2012). Experimental study of aortic flow in the ascending aorta via particle tracking velocimetry. Exp Fluids.

[60] Pedocchi F, Martin JE, García MH (2008). Inexpensive fluorescent particles for large-scale experiments using particle image velocimetry. Exp Fluids.

[61] Thielicke W, Sonntag R (2021). Particle image velocimetry for MATLAB: Accuracy and enhanced algorithms in PIVlab. J Open Res Softw..

[62] Qiao Y, Luo K, Fan J (2023). Heat transfer mechanism in idealized healthy and diseased aortas using fluid–structure interaction method. Biomech Model Mechanobiol.

[63] Luan J, Qiao Y, Mao L, Fan J, Zhu T, Luo K (2023). The role of aorta distal to stent in the occurrence of distal stent graft-induced new entry tear: a computational fluid dynamics and morphological study. Comput Biol Med.

[64] Szykiedans K, Credo W, Osiński D (2017). Selected mechanical properties of PETG 3-D prints. Proc Eng.

[65] Chakraborty P, Balachandar S, Adrian RJ (2005). On the relationships between local vortex identification schemes. J Fluid Mech.

