# Supplementary material for: Integrated particle image velocimetry and fluid–structure interaction analysis for patient-specific abdominal aortic aneurysm studies
Source: Biomed Eng Online. 2023 Dec 3;22:113. doi: 10.1186/s12938-023-01179-8 (PMC10693692; doi:10.1186/s12938-023-01179-8)
Supplement: Supplementary file 1 — Additional file 1: Table S1. Grid parameters and the total number of the CFD and mechanical domains. Table S2. Mesh sensitivity results for constant flow at 11.5 L/min flow rate condition. Table S3. Mesh sensitivity results for pulsatile flow at 60 BPM condition. Table S4. Solver parameters for the transient FSI run. Figure S1. Total wall displacement under constant flow (11.5 L/min flowrate) condition (Coarse-Chosen-Fine Mesh). Figure S2. Velocity at cut plane under constant flow (11.5 L/min flow rate) condition. a Fine, b chosen, c coarse mesh. Figure S3. Comparison of maximum velocity of the domain vs time, pulsatile flow at 60 BPM condition. Figure S4. Comparison of space averaged velocity over time for cut-plane, pulsatile flow at 60 BPM condition. Figure S5. Time average wall shear stress (TAWSS), pulsatile flow at 60 BPM condition. Figure S6. Time averaged displacement (TAD), pulsatile flow at 60 BPM condition. Figure S7. Space averaged Von-Mises stress at wall, pulsatile flow at 60 BPM condition. [file 12938_2023_1179_MOESM1_ESM.docx]

Table S1 Grid parameters and the total number of the CFD and mechanical domains

| **Parameter / Mesh Resolution** | **Coarse** | **Chosen** | **Fine** |
| --- | --- | --- | --- |
| **FEA Size [mm]** | 1.5 | 1.25 | 1 |
| **Node, Mechanical** | 408,129 | 546,785 | 693,574 |
| **Elem, Mechanical** | 224,523 | 303,469 | 384,867 |
| **CFD Global Size [mm]** | 1.5 | 1.25 | 1 |
| **# Boundary Layer** | 3 | 5 | 8 |
| **Max. Thickness [mm]** | 0.75 | 0.5 | 0.35 |
| **Node, CFD** | 92,090 | 171,954 | 386,674 |
| **Elem, CFD** | 362,047 | 664,527 | 1,176,759 |

Table S2 Mesh sensitivity results for constant flow at 11.5 L/min flow rate condition

| **Parameter / Mesh Resolution** | **Coarse** | **Chosen** | **Fine** |
| --- | --- | --- | --- |
| **Max. Velocity [m/s]** | 2.958 (2.4%) | 3.031 (-%) | 3.061 (1.0%) |
| **Max. Total Deformation [mm]** | 1.881 (8.0%) | 2.047 (-%) | 2.055 (0.5%) |
| **Max. Von Mises Stress [Pa]** | 92,285 (1.2%) | 93,375 (-%) | 104,960 (11.0%) |
| **Max. Von Mises Strain [m/m]** | 0.133 (1.5%) | 0.135 (-%) | 0.151 (10.8%) |
| **Max. Wall Shear Stress [Pa]** | 88.906 (49.7%) | 176.712 (-%) | 174.945 (1.0%) |
| **Area Ave. Wall Shear [Pa]** | 6.502 (27.1%) | 8.914 (-%) | 9.765 (8.7%) |

Table S3 Mesh sensitivity results for pulsatile flow at 60 BPM condition

| **Parameter / Mesh Resolution** | **Coarse** | **Chosen** | **Fine** |
| --- | --- | --- | --- |
| **Max. Velocity [m/s]** | 1.831 (4.0%) | 1.908 (-%) | 1.942 (1.8%) |
| **Max. Displacement [mm]** | 3.061 (8.1%) | 3.329 (-%) | 3.481 (4.4%) |
| **TAWSS [Pa]** | 12.988 (16.8%) | 15.6015 (-%) | 18.691 (16.5%) |
| **Max. TAD [mm]** | 0.574 (3.7%) | 0.596 (-%) | 0.615 (3.1%) |
| **Max. TAVMS [kPa]** | 10.855 (2.6%) | 11.143 (-%) | 11.265 (1.1%) |


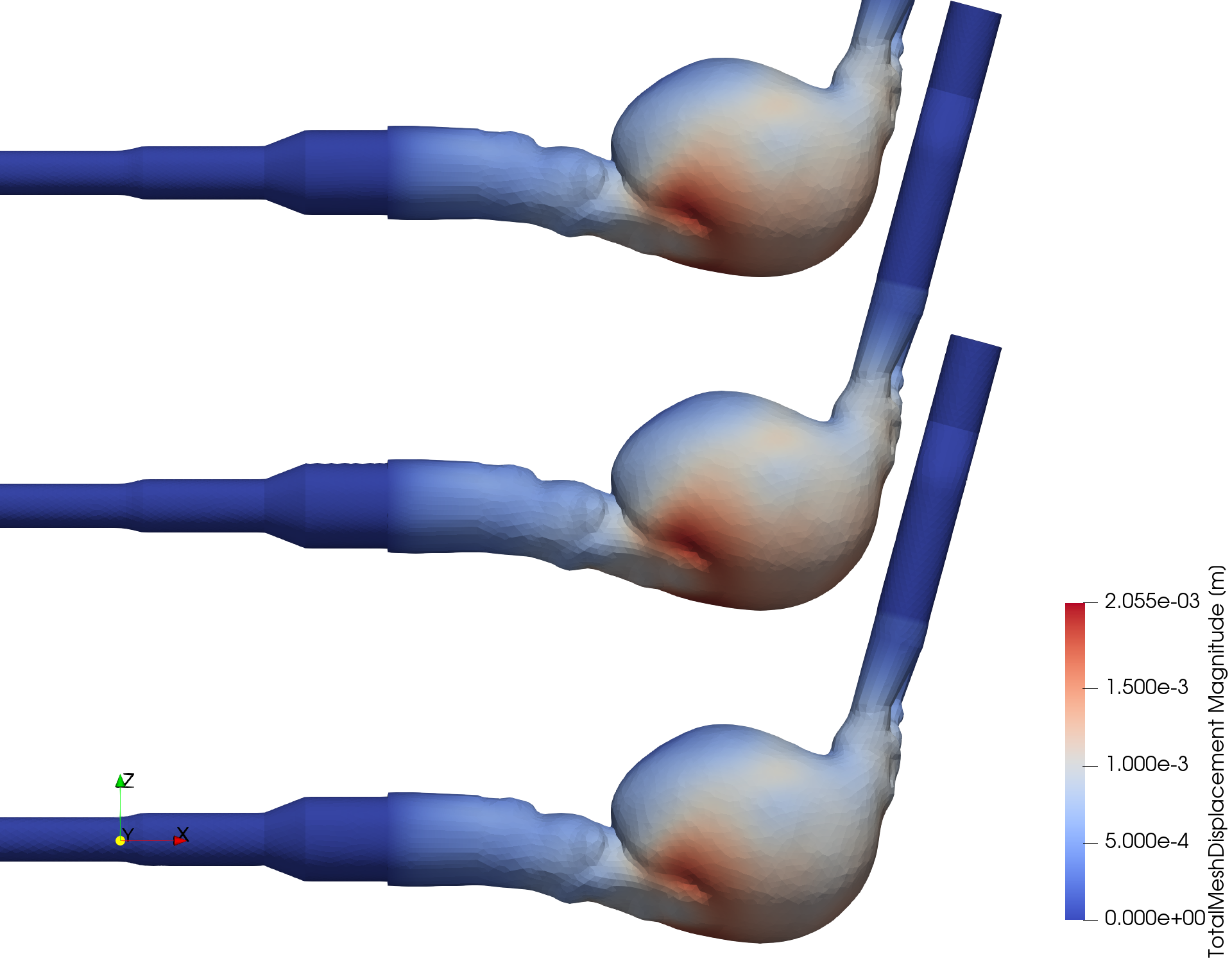


Fig. S1 Total wall displacement under constant flow (11.5 L/min flowrate) condition (Coarse-Chosen-Fine Mesh)


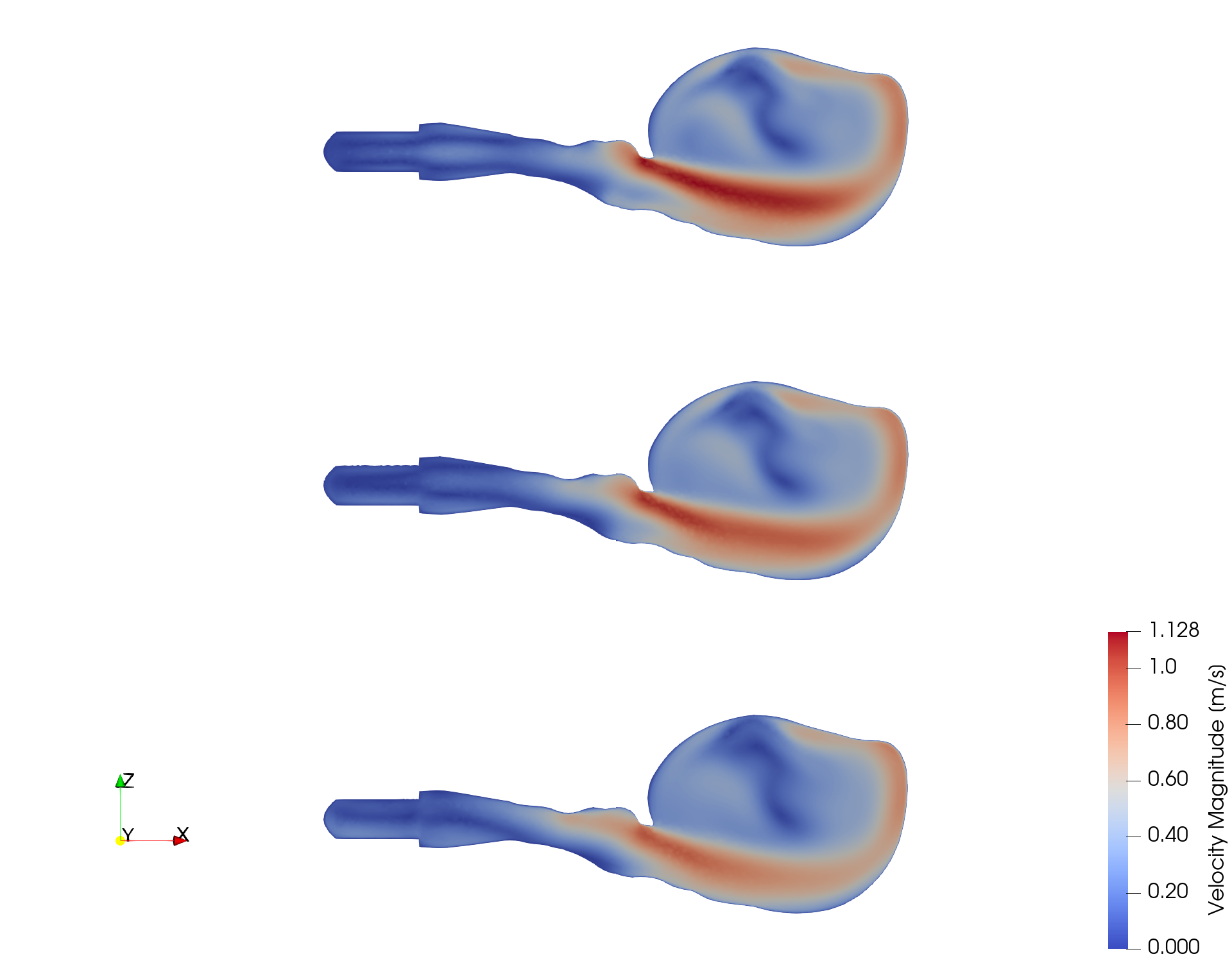


Fig. S2 Velocity at cut plane under constant flow (11.5 L/min flow rate) condition (a) fine (b) chosen (c) coarse mesh


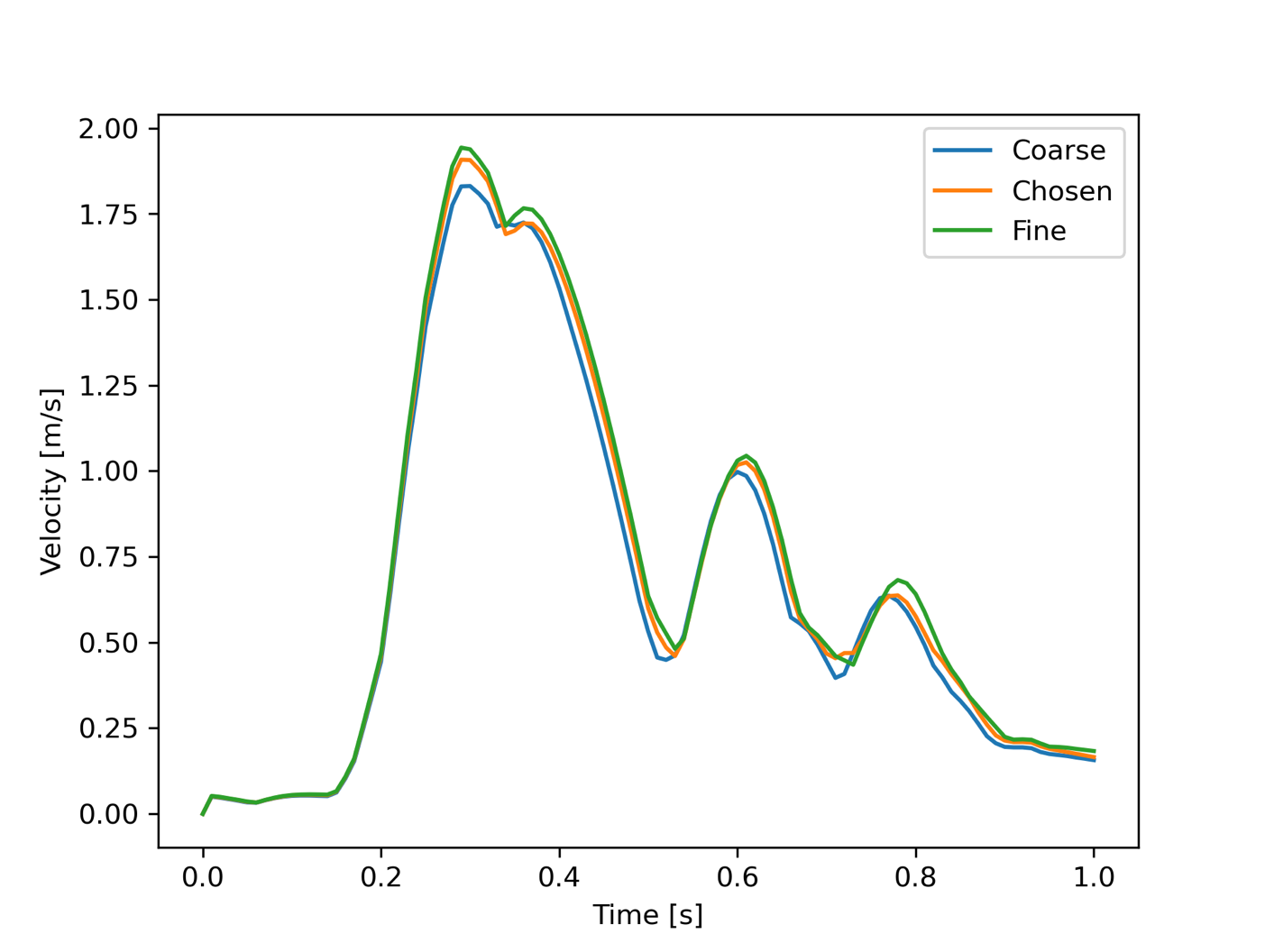


Fig. S3 Comparison of maximum velocity of the domain vs time, pulsatile flow at 60 BPM condition


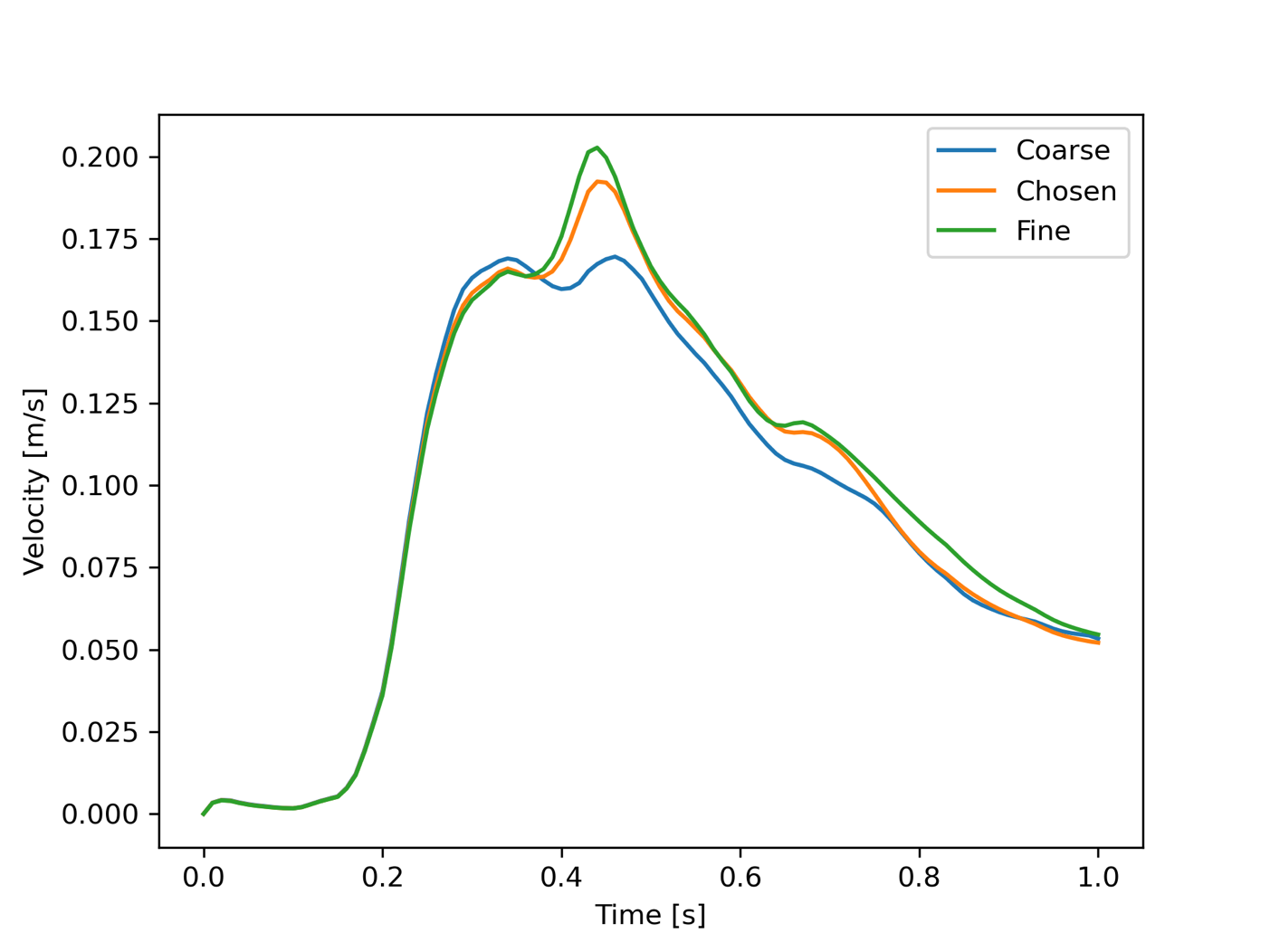


Fig. S4 Comparison of space averaged velocity over time for cut-plane, pulsatile flow at 60 BPM condition


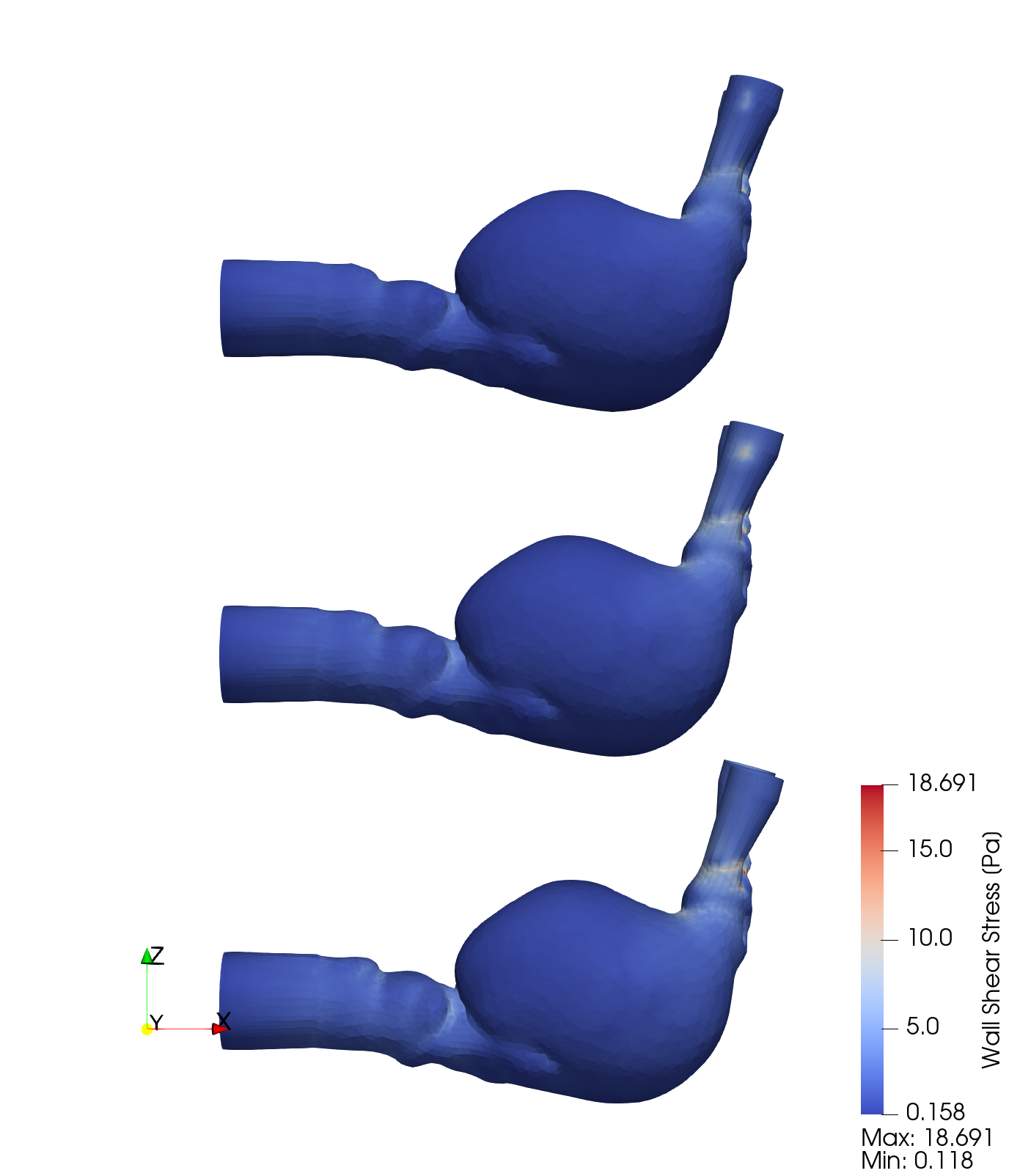


Fig. S5 Time average wall shear stress (TAWSS), pulsatile flow at 60 BPM condition


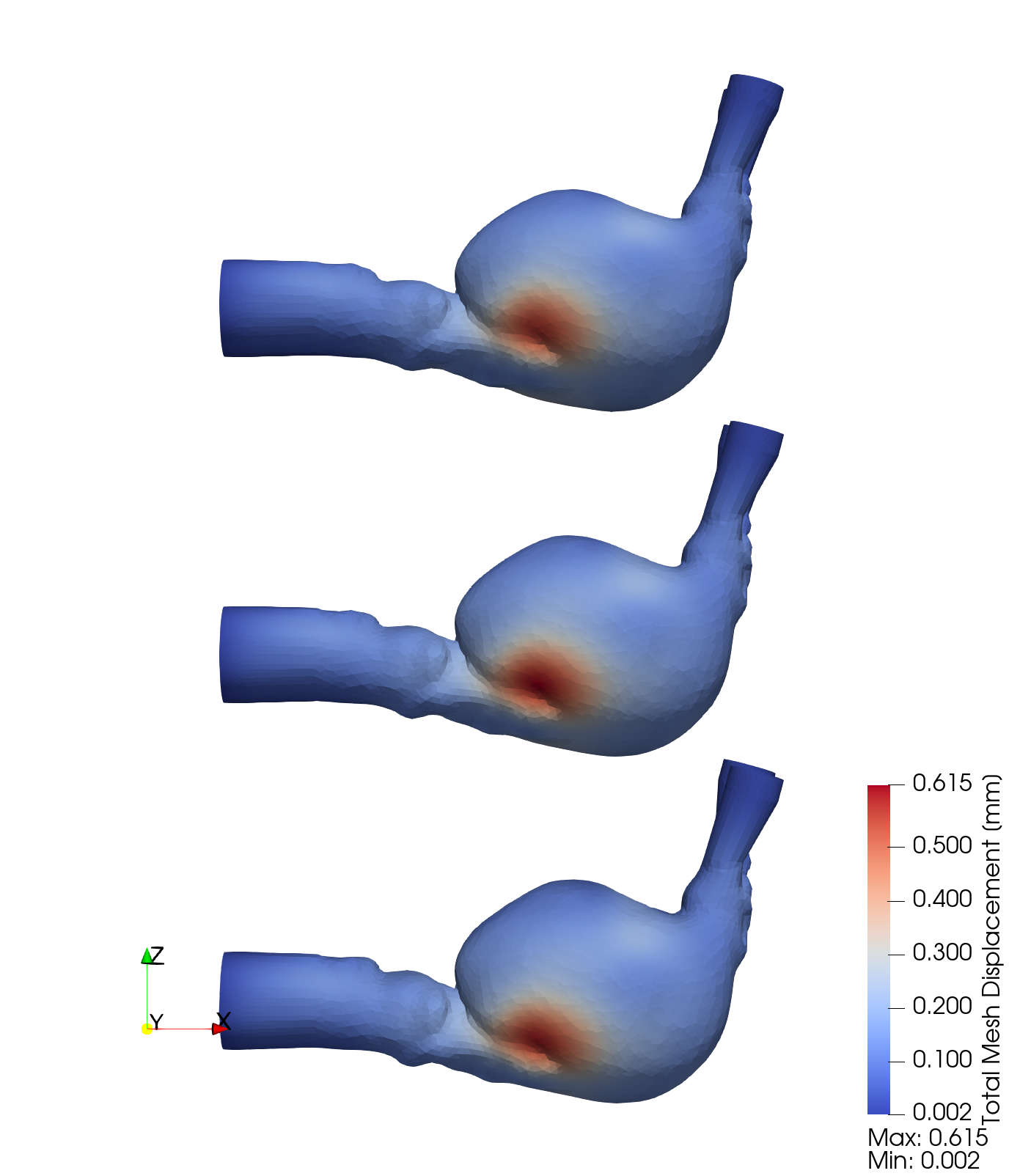


Fig. S6 Time averaged displacement (TAD), pulsatile flow at 60 BPM condition


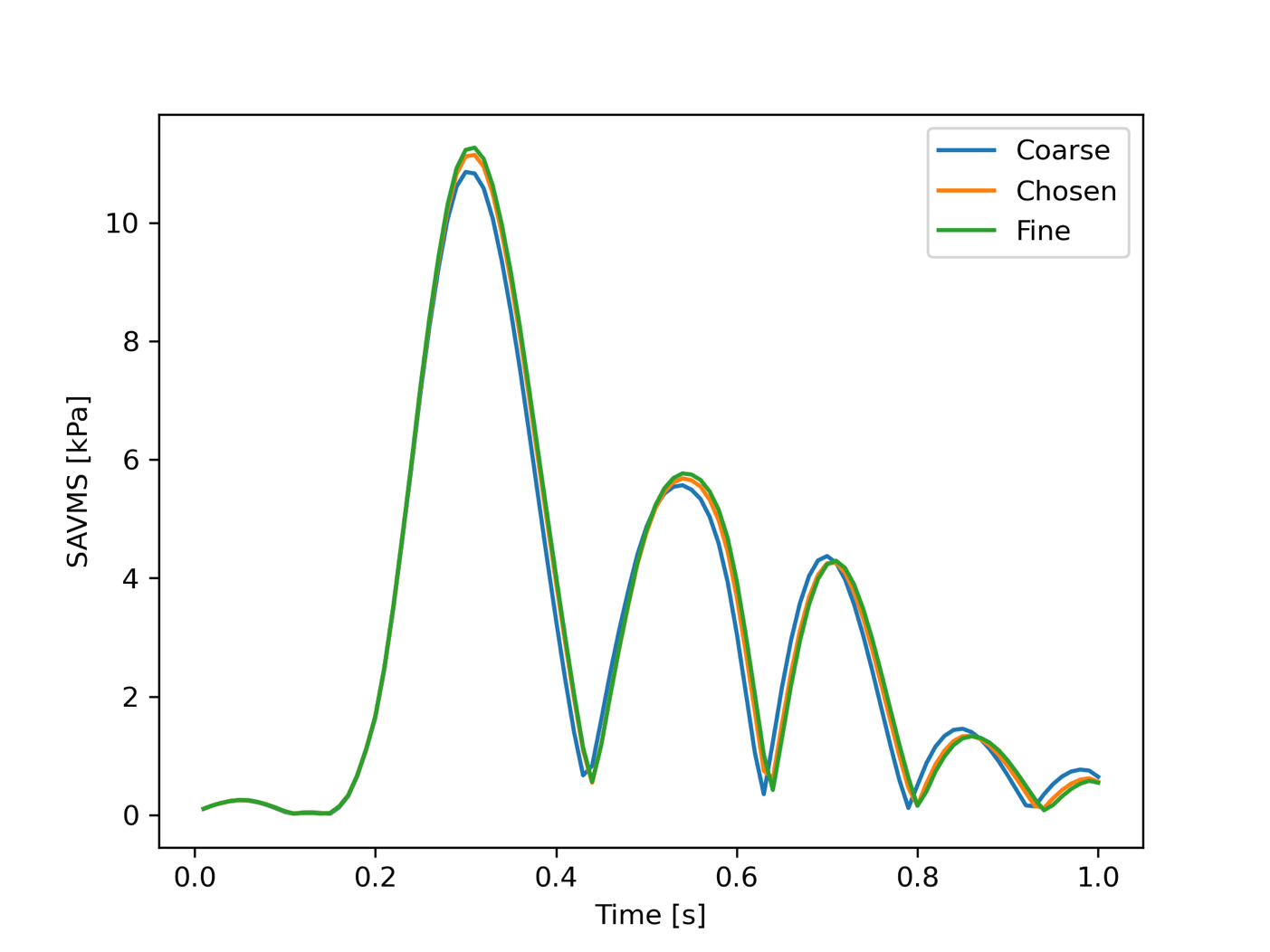


Fig. S7 Space averaged Von-Mises stress at wall, pulsatile flow at 60 BPM condition

Table S4 Solver parameters for the transient FSI run

| **Solver** | **Solver Parameter** | **Value** |
| --- | --- | --- |
| **Ansys System Coupling** | Coupling time step [s] | 0.01 |
|  | Coupling force transfer convergence criteria | 0.01 |
|  | Under relaxation factor – Force | 0.5 |
|  | Coupling displacement transfer convergence criteria | 0.001 |
|  | Under relaxation factor – Displacement | 1.0 |
|  | Minimum iterations | 10 |
|  | Maximum iterations | 20 |
| **Ansys CFX** | Time step size [s] | 0.01 |
|  | Minimum coefficient loops | 2 |
|  | Maximum coefficient loops | 10 |
|  | Convergence Criteria > Residual Type | RMS |
|  | Convergence Criteria > Residual Target | 1e-4 |
|  | Conservation Target | 0.01 |
|  | Advection scheme | High Res. |
|  | Transient scheme | Second order backward Euler |
|  | Turbulence numberics | First order |
| **Ansys Mechanical** | Large deflection effects | Turned on |
